# The *ATXN2-SH2B3* locus is associated with peripheral arterial disease: an electronic medical record-based genome-wide association study

**DOI:** 10.3389/fgene.2014.00166

**Published:** 2014-06-25

**Authors:** Iftikhar J. Kullo, Khader Shameer, Hayan Jouni, Timothy G. Lesnick, Jyotishman Pathak, Christopher G. Chute, Mariza de Andrade

**Affiliations:** ^1^Division of Cardiovascular Diseases, Mayo ClinicRochester, MN, USA; ^2^Biomedical Statistics and Informatics, Health-Related Sciences, Mayo ClinicRochester, MN, USA

**Keywords:** genome-wide association study, peripheral arterial disease, ankle-brachial index, electronic medical records, biorepository

## Abstract

**Objectives:** In contrast to coronary heart disease (CHD), genetic variants that influence susceptibility to peripheral arterial disease (PAD) remain largely unknown.

**Background:** We performed a two-stage genomic association study leveraging an electronic medical record (EMR) linked-biorepository to identify genetic variants that mediate susceptibility to PAD.

**Methods:** PAD was defined as a resting/post-exercise ankle-brachial index (ABI) ≤0.9 or ≥1.4 and/or history of lower extremity revascularization. Controls were patients without history of PAD. In Stage I we performed a genome-wide association analysis adjusting for age and sex, of 537, 872 SNPs in 1641 PAD cases (66 ± 11 years, 64% men) and 1604 control subjects (61 ± 7 year, 60% men) of European ancestry. In Stage II we genotyped the top 48 SNPs that were associated with PAD in Stage I, in a replication cohort of 740 PAD cases (70 ± 11 year, 63% men) and 1051 controls (70 ± 12 year, 61% men).

**Results:** The SNP rs653178 in the *ATXN2-SH2B3* locus was significantly associated with PAD in the discovery cohort (*OR* = 1.23; *P* = 5.59 × 10^−5^), in the replication cohort (*OR* = 1.22; 8.9 × 10^−4^) and in the combined cohort (*OR* = 1.22; *P* = 6.46 × 10^−7^). In the combined cohort this SNP remained associated with PAD after additional adjustment for cardiovascular risk factors including smoking (*OR* = 1.22; *P* = 2.15 × 10^−6^) and after excluding patients with ABI > 1.4 (*OR* = 1.24; *P* = 3.98 × 10^−7^). The SNP is in near-complete linkage disequilibrium (LD) (*r*^2^ = 0.99) with a missense SNP (rs3184504) in *SH2B3*, a gene encoding an adapter protein that plays a key role in immune and inflammatory response pathways and vascular homeostasis. The SNP has pleiotropic effects and has been previously associated with multiple phenotypes including myocardial infarction.

**Conclusions:** Our findings suggest that the *ATXN2-SH2B3* locus influences susceptibility to PAD.

## Introduction

Peripheral arterial disease (PAD) affects nearly 10 million people in the US and more than 200 million people worldwide (Hirsch et al., 2001; Fowkes et al., 2013). PAD is associated with significant mortality and morbidity, underscoring the need to discover genetic variants that mediate susceptibility to this disease (Leeper et al., 2012). In contrast to coronary heart disease (CHD), genetic variants that influence susceptibility to PAD remain unknown. A genome-wide association study (GWAS) of smoking quantity revealed a variant in *CHRNA3* that was associated with PAD and lung cancer (Thorgeirsson et al., 2008).

Repositories of DNA from patients seen in the clinical setting and linked to the electronic medical record (EMR) systems can be leveraged to conduct genotyping or sequencing studies to identify genetic variants associated with human diseases and related quantitative traits. Extensive clinical data residing in the EMR can be leveraged for high-throughput phenotyping of medically relevant traits (Kullo et al., 2010). Such an approach may reduce the time, effort, and cost involved in conducting genomic studies to identify disease susceptibility loci.

The Electronic Medical Records and Genomics (eMERGE) consortium (McCarty et al., 2011) was created to develop and implement approaches for leveraging biorepositories linked to the EMR for large-scale genomic research, including but not limited to GWAS, sequencing, and structural variation (Kho et al., 2011). We undertook a GWAS of PAD cases and controls identified from the EMR using a two-stage study design. In Stage I we performed a GWAS of 1641 PAD cases and 1604 controls, and in Stage II we attempted replication of the top significant SNPs in an independent sample of 740 PAD cases and 1051 controls.

## Materials and methods

### Study participants

All participants gave written informed consent for participation in the study and the use of their data for future research. The Institutional Review Board of the Mayo Clinic approved the study protocol.

### Ascertainment of PAD cases and controls

The PAD patients were recruited from the non-invasive vascular laboratory at the Mayo Clinic Rochester, MN, based on the following criteria: (1) an ankle brachial index (ABI) of ≤0.9 at rest or 1 min after exercise, along with an abnormal continuous wave Doppler signal in one of the lower extremity arteries; (2) history of lower extremity revascularization if the ABI was normal; and (3) ABI ≥ 1.4 or ankle systolic BP > 250 mm Hg, representing poorly compressible arteries. Exclusion criteria included PAD secondary to vasculitis, radiation to the abdomen or lower extremities, trauma to a lower extremity artery, thrombophilia, and arterial thrombosis. Controls were identified from patients referred to the Cardiovascular Health Clinic for exercise ECG to screen for cardiovascular disease. We excluded patients who had a positive exercise ECG, were younger than age 50, or had an abnormal ABI or history of PAD. A proportion (60%) of the subjects who underwent exercise ECG also underwent measurement of ABI. The prevalence of an abnormal ABI in patients who had a negative stress ECG was <1%.

Patient-level data elements in the Mayo EMR included demographics, outpatient visits and hospitalizations, providers, diagnosis and procedure codes, and results of non-invasive lower extremity arterial evaluation. Birth date, race, sex, and ethnicity were obtained from the demographic database; the categories for race were “White,” “Black or African American,” “Hispanic,” “Asian/Pacific Islander,” “American Indian/Alaskan Native,” “Others,” and “Unknown.”

### Stage-I: high-density genotyping of discovery cohort

Genotyping was performed using the Illumina 660W-Quad BeadChip at the Center for Genotyping and Analysis at the Broad Institute, Cambridge, MA. This platform consists of 561,490 SNPs and 95,876 intensity-only probes. In addition to 3347 patient DNA samples, 58 blind duplicates, and 37 Coriell controls were genotyped. The Coriell controls include 1 trio (3 unique samples) that was duplicated on each plate. Genotyping calls were made using BeadStudio version 3.3.7 (2010).

Analysis tools used for quality control (QC) procedures included Illumina BeadStudio (2010), PLINK (Purcell et al., 2007), *R* (The R Development Core Team, 2007), STRUCTURE (Pritchard et al., 2000), and Eigenstrat in the Eigensoft package (Price et al., 2006). Data were cleaned using the QC pipeline developed by the eMERGE Genomics Working Group (Turner et al., 2011). This process includes evaluation of sample and marker call rate, gender mismatch and anomalies, duplicate and HapMap concordance, batch effects, Hardy-Weinberg equilibrium, sample relatedness, and population stratification. The data from all the patients, in addition to the HapMap II populations, were evaluated for population structure/substructure using Eigenstrat (Price et al., 2006). Of the 3347 unique samples, 3336 passed genotyping QC (see Supplementary Data and Figures S1–S3).

### Stage-II: genotyping of lead SNPs in the replication cohort

The replication cohort consisted of 744 (470 males and 274 females) patients who had PAD based on the criteria listed above and 1053 (645 males and 408 females) controls with no prior history of PAD. The top 48 SNPs associated with PAD in the discovery cohort were genotyped using an Illumina custom genotyping panel with primers and probes from Assay-by-Design (Applied Biosystems, Foster City, CA). Custom capture and genotyping was performed at Mayo Clinic's Genotyping Core lab/Genotyping Shared Resource Lab.

Standard QC procedures were applied including evaluation of sample and marker call rate, HapMap concordance, and Hardy-Weinberg in controls only. We excluded six patients with low call rates (<95%). Of the 48 SNPs selected for replication, one (rs7900716) had a low call rate. All the 47 remaining SNPs had call rates >99% and Hardy-Weinberg *P*-value > 0.05 in the controls.

### Statistical analyses

Statistical analyses were conducted using SAS v. 9.3 {SAS Institute Inc., Cary, NC} and PLINK v1.07 (Purcell et al., 2007), and plots were created using *R* v2.11.0 (The R Development Core Team). Descriptive analyses were performed for the covariates and outcome variables using *t*-tests for continuous variables and chi-square tests for discrete variables. To adjust for population stratification, we used principal components to identify outliers in the study cohort (Price et al., 2006). Quantile-quantile (QQ) plots of observed –log_10_ *P*-values for PAD association versus the expected –log_10_ *P*-values under the null hypothesis of no association were generated to display the potential significant associations and to calculate the genomic inflation factor λ and to check for over dispersion of the test statistics. For each locus, we determined the set of HapMap SNPs in linkage disequilibrium (LD) (*r*^2^ > 0.5) with the most significantly associated SNP. We then bounded the associated interval by the flanking HapMap recombination hotspots. These windows are likely to contain the causal variants explaining the associations. We used logistic regression analyses that adjusted for age and sex to identify the SNPs associated with PAD case/control status in the discovery, replication, and combined sets. All analyses were forced to test the same allele as the original sample. We performed sensitivity analyses by including additional adjustment variables for smoking, CHD, statin use, diastolic and systolic blood pressure, and diabetes. Since the additional adjustment variables did not have a qualitative impact on the final inferences, the results are not shown.

### Functional annotation of the lead SNP

Data for the SNP rs3184504 (c.784T>C), which is in nearly complete LD with the most significant SNP, were obtained from the Exome Variant Server. The impact of the variant was assessed using SIFT (Ng and Henikoff, 2003), PoplyPhen2 (Adzhubei et al., 2010), and conservation based measures such as PhastCons (Siepel et al., 2005), GRANTHAM (Grantham, 1974), and GERP (Cooper et al., 2005) scores. We performed Gene Ontology (GO) term enrichment analysis of *SH2B3* using first-degree interacting partners that were obtained from the protein-protein interaction database “STRING” (http://www.string-db.org). To understand the impact of SNP rs3184504 on protein structure, we performed a molecular dynamics simulation using GROMACS v4.5.7 (http://www.gromacs.org/) of the pleckstrin homolog (PH) domain of the SH2B3 protein where the SNP is localized.

## Results

### Discovery

**Figure 1:**
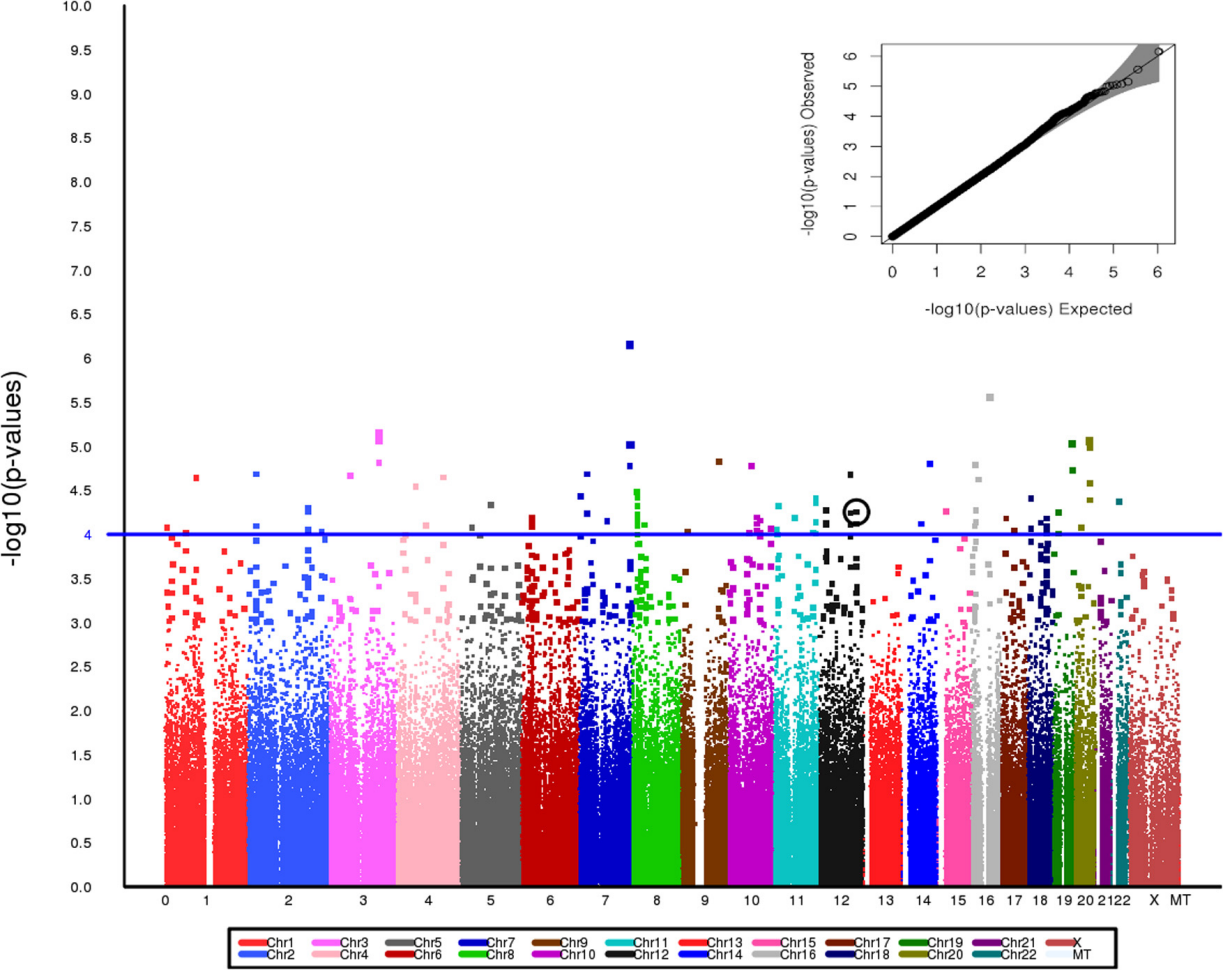
**Manhattan plot for the Stage I discovery cohort showing genome wide *P*-value distribution and the corresponding Q-Q plot (inset)**. SNP rs653178 is circled. A line at *p* = 1 × 10^−4^ is included on the Manhattan plot to provide a visual reference for *p*-values across the chromosomes.

After exclusions based on QC, including removal of related individuals and those of non-European Ancestry, a total of 3245 individuals—1641 PAD subjects and 1604 controls—were included in the analyses. No evidence of population stratification was found and therefore correction for population stratification was not needed in the analyses. Since the estimate of λ was 1.0, the test statistics showed no significant over dispersion. The study population demographic and clinical characteristics by case-control status are presented in Table 1. Among PAD cases, 64.3% were men, while among the controls, 60.3% were men. The mean age of the PAD patients was higher than the mean age of the control patients (65.7 years vs. 60.8 years) (Table 1). Assuming an additive genetic model and adjusting for age and sex, 60 SNPs were associated with PAD at *P* < 1 × 10^−4^. Figure 1 presents a Manhattan plot of the *P*-values. Of these 60 SNPs, 48 were selected for replication based on Illumina designability score, LD, and minor allele frequency (MAF) in controls (see Supplementary Data for details).

**Table 1:** **Participant characteristics**.

	**Discovery cohort**		**Replication cohort**	
	**Cases (*n* = **1641**)**	**Controls (*n* = **1604**)**	**Cases (*n* = **740**)**	**Controls (*n* = **1051**)**
Men, *n* (%)	1055 (64.3)	968 (60.3)	468 (63.2)	643 (61.2)
Age, years	65.7 ± 10.68	60.8 ± 7.41^‡^	70.6 ± 11.60	70.2 ± 12.42
European ancestry, *n* (%)	1547 (94.3)	1512 (94.3)	721 (97.4)	1023 (97.3)
“Ever” smoker, *n* (%)	1322 (80.5)	963 (60.1)	632 (85.4)	641 (61.0)
ABI (pre-exercise)	0.72 ± 0.25	1.1 ± 0.07^‡^	0.79 ± 0.30	1.07 ± 0.16^‡^
ABI (post-exercise)	0.54 ± 0.25	1.1 ± 0.12^‡^	0.56 ± 0.28	1.03 ± 0.19^‡^
Hypertension, *n* (%)	1358 (82.8)	843 (52.6)^‡^	583 (78.8)	634 (60.3)^‡^
Type 2 diabetes, *n* (%)	507 (30.9)	141 (8.8)^‡^	225 (30.4)	126 (12.0)^‡^
Statin use, *n* (%)	774 (49.2)	398 (24.8)^‡^	532 (72.0)	326 (61.1)^‡^
CHD, *n* (%)	903 (55)	251 (15.6)^‡^	483 (65.3)	235 (22.4)^‡^

Continuous traits are depicted as mean ± standard deviation and categorical traits as count (percent); ABI, ankle-brachial index; CHD, coronary heart disease.

‡ P < 0.001 for differences between PAD cases and controls.

### Replication

Characteristics of participants in the discovery and replication cohorts are presented in Table 1. The allele C of the intronic SNP rs653178 at the *ATXN2-SH2B3* locus on chromosome 12 was present more frequently in PAD cases (52%) than in controls (47%) with a resulting odds ratio (OR) of 1.23 (95% CI, 1.11–1.36, *P* = 5.59 × 10^−5^) in the discovery cohort (Table 2). In the replication cohort, the OR was 1.25 (95% CI, 1.10–1.40, *P* = 8.94 × 10^−4^) and in the combined sample, the OR was 1.22 (95% CI, 1.13–1.32, *P* = 6.46 × 10^−7^) (Table 2). The lead SNP rs653178 is in strong LD (*r*^2^ = 0.99) with a missense SNP (rs3184504) in *SH2B3*, an adapter protein that plays a key role in immune and inflammatory response pathways and vascular homeostasis (Devalliere and Charreau, 2011; Devalliere et al., 2012). A locus specific visualization of lead variants associated with PAD is provided in the Supplementary Data (Figure S4).

**Table 2:** **Association of rs653178 with PAD in discovery, replication, and combined cohorts, after adjustment for age and sex in logistic regression models**.

**Cohort**	**PAD (*n*)**	**Controls (*n*)**	Risk allele (frequency^*^)	**OR (95% CI)**	***P*-value**
Discovery	1641	1604	C (0.469)	1.23 (1.11,1.36)	5.59 × 10^−5^
Replication	740	1051	C (0.475)	1.25 (1.10,1.40)	8.90 × 10^−4^
Combined	2381	2655	C (0.469)	1.22 (1.13,1.32)	6.46 × 10^−7^

CI, confidence interval; OR, odds ratio; PAD, peripheral disease.

* Controls.

Two additional SNPs rs11726269 (intronic region of *MAPK10*) and rs131408 (intergenic region between *LOC388882* and *IGLL1*) were significant at *P* < 0.05 in the replication cohort with similar direction of effect. However the *P* values exceeded the Bonferroni threshold for testing 48 SNPs (see Supplementary Table S1). The two most significant SNPs in the discovery cohort, rs7795096 in *PRKAG2* on chromosome 7 and rs2587888 in *GNAO1* on chromosome 16, did not replicate.

## Structural and functional implications of the W262R variant in the SH2B3 protein

Our analyses indicate that *SH2B3* encodes a multi-functional protein involved in diverse molecular pathways. Comparative protein sequence analyses using wild type and mutant sequences indicated that that rs3184504 leads to substitution of tryptophan with arginine (W262R) thereby introducing a new cAMP phosphorylation site in the PH domain of SH2B3 (see Supplementary Data and Figure S5). The PH domain in SH2B3 is important for lipid binding, membrane tethering and protein-protein interactions. GO terms (Ashburner et al., 2000) that are enriched among proteins interacting with *SH2B3* include blood coagulation; wound healing, and cell signaling events (see Supplementary Data, Table S2 and Figure S6). Conservation measures like Genomic Evolutionary Rate Profiling (GERP: 2.97) and phastCons (posterior probability: 0.159) suggest the variant is marginally conserved. Effect prediction analysis using Variant Effect Predicator (McLaren et al., 2010) indicate the variant as tolerant (SIFT: score = 1) benign (PolyPhen-2; score = 0.0), and moderately radical (GRANTHAM; score = 101). Molecular dynamic simulation suggested that the mutation in the PH domain of the SH2B3 results in structural perturbations and conformational changes (see Supplementary Data; Figures S7 and S8).

## Discussion

A better understanding of the genetic basis of PAD is required to improve risk stratification and identify new pathophysiologic pathways and drug targets. Conventional linkage and association approaches have failed to identify replicable susceptibility loci for PAD (Leeper et al., 2012) and the genome-wide association approach is currently the most promising design to uncover such loci. Heritable factors contribute to the risk of developing PAD. In the large population-based Swedish Twin Registry (Wahlgren and Magnusson, 2011), the odds ratio of having PAD in persons whose twin had PAD compared with persons whose twin did not have PAD was 17.7 (95% CI, 11.7–26.6) for monozygotic twins and 5.7 (95% CI, 4.1–7.9) for dizygotic twins. In a large case control study we found that family history of PAD was associated with doubling the odds of the presence of PAD (Khaleghi et al., 2014). Heritability estimates for ABI have varied from 0.21 (Kullo et al., 2006; Murabito et al., 2006) to 0.48 (Carmelli et al., 2000). In spite of evidence supporting the presence of heritable contribution to PAD, little is known about the genetic determinants of PAD.

In the present study, the SNP most strongly associated with PAD was an intronic SNP rs653178 in *ATXN2* on chromosome 12q24-12q24.1. This SNP is in near-complete LD with a missense SNP in SH2B adaptor protein 3 gene (*SH2B3)* (rs3184504; *r*^2^ = 0.99) that is likely the causal SNP. The SNP in *SH2B3* results in a substitution of tryptophan (large size and aromatic side chain) by arginine (large size and basic side chain) that induces changes in the structure and hydrophilic properties of the pleckstrin homology domain. This may result in altered lipid binding and protein–protein interactions as indicated by our molecular dynamics analyses. The variant also introduces a new phosphorylation site in the pleckstrin homology domain which may influence signaling pathways mediated by SH2B3. The SNP rs3184504 exhibits significant pleiotropic effects and has been implicated in immunological disorders, cardiovascular diseases (Gudbjartsson et al., 2009) and hematologic traits such as platelet count, mean-platelet volume (Gieger et al., 2011) and eosinophil count (Barrett et al., 2009). A summary of disease/trait associations of rs3184504 and rs653178 in the *ATXN2-SH2B3* locus is provided in the Supplement (Table S3).

The pleiotropic nature of *SH2B3* may be due to its role in immune and inflammatory signaling pathways including erythropoietin, cytokine receptor-mediated and integrin signaling (20). The protein also regulates hematopoietic cell lineage and endothelial cells, and influences adhesion and migration of platelets by modulating actin cytoskeleton organization (Takizawa et al., 2010; Gieger et al., 2011; Devalliere et al., 2012; Shameer et al., 2014). *SH2B3* is also involved in platelet production via megakaryocyte development; mice lacking *SH2B3* (*L*nk/SH2B3^−/−^) (Kwon et al., 2009) have altered platelet function and thrombus development (Tong et al., 2005). The relatively high frequency of this SNP in the general population is speculated to be due to a protective effect against bacterial infection (Zhernakova et al., 2010). We (Ding and Kullo, 2011) and others (Pickrell et al., 2009) have previously demonstrated that the SNP may have been subject to natural selection.

Two GWAS in European ancestry cohorts have reported variants associated with PAD. Thorgeirsson et al (Thorgeirsson et al., 2008) found a common variant in the nicotinic acetylcholine receptor gene cluster on chromosome 15q24 to affect nicotine dependence, smoking quantity, and the risk of PAD and lung cancer. A synonymous SNP (rs1051730) within the cholinergic receptor nicotinic alpha 3 gene (*CHRNA3*) was significantly associated with PAD (*OR* = 1.19). In a meta-analysis (Murabito et al., 2012) of GWAS for ABI consisting of 21 population-based cohort studies and 41,692 participants of European ancestry among whom 3409 participants had PAD (ABI < 0.90), six SNPs were associated (*P* = 1 × 10^−6^) with PAD, but none at a genome-wide significance level. The *ATXN2*-*SH2B3* locus was not associated with PAD in this study. One possible explanation may be the differences in case ascertainment, the present study including symptomatic PAD patients from the clinical setting whereas in the meta-analyses by Murabito et al, most individuals had undergone ABI measurement as part of prospective cohort studies. Koriyama et al. (2010) found the *OSBPL10* locus to be associated with PAD in a Japanese cohort. We assessed the strength of association of these SNPs in our dataset and found that the 9p21 variant and the *OSBPL10* variants were not associated, whereas the *CHRNA3* variant was weakly (*P* = 1 × 10^−3^) associated with PAD case status.

In conclusion, our findings suggest that SNP rs653178 in the *ATXN2-SH2B3* locus is associated with clinically defined PAD. The SNP is in near complete LD with rs3184504, a non-synonymous SNP in *SH2B3*, a gene implicated in immune, inflammatory, and hematopoietic pathways. This SNP is emerging as a key pleiotropic genetic variant influencing multiple cardiovascular traits. Our findings motivate additional investigation of this locus including sequencing, gene expression and drug targeting studies as well as studies in experimental animals.

## Sources of funding

This work was supported by grants HG-04599 and HG-06379 from the National Human Genome Research Institute (NHGRI), Bethesda, MD. The eMERGE Network was initiated and funded by NHGRI, with additional funding from National Institute of General Medical Sciences (NIGMS), Bethesda, MD.

### Conflict of interest statement

The authors declare that the research was conducted in the absence of any commercial or financial relationships that could be construed as a potential conflict of interest.
