# Supplementary material for: The ATXN2-SH2B3 locus is associated with peripheral arterial disease: an electronic medical record-based genome-wide association study
Source: Front Genet. 2014 Jun 25;5:166. doi: 10.3389/fgene.2014.00166 (PMC4070196; doi:10.3389/fgene.2014.00166)
Supplement: Supplementary file 1 [file DataSheet1.PDF]

Supplementary Data:

The *ATXN2-SH2B3* locus is Associated with Peripheral Arterial Disease: An Electronic Medical Record-Based Genome-Wide Association Study

Authors:
Iftikhar J. Kullo, MD;[*^] Khader Shameer, PhD;[*] Hayan Jouni, MD;[*] Timothy G. Lesnick, MS;[†] Jyotishman Pathak, PhD;[†] Christopher G. Chute, MD, DrPh;[†] and Mariza de Andrade, PhD[†]

Affiliations:
* Division of Cardiovascular Diseases, Mayo Clinic, 200 First Street SW, Rochester, MN 55905;
† Biomedical Statistics and Informatics, Health-Related Sciences, Mayo Clinic, 200 First Street SW, Rochester, MN 55905

Short title: Kullo et. al – A Genome-Wide Association Study for PAD

^Corresponding author:
Iftikhar J. Kullo, MD
200 First Street SW
Rochester, MN 55905
Fax: 507-538-6418
Phone: 507-284-9049
Email: Kullo.iftikhar@mayo.edu

Discovery Phase

Quality control:. Of the 3336 unique samples, 35 subjects were related (9 unexpected parent-offspring pairs, 17 unexpected full sibling pairs, 12 unexpected grandparent-grandchild/avuncular/half-sib pairs, and 8 unexpected duplicates/monozygotic twins).(Purcell et al.) Twenty-five subjects had chromosomal anomalies: 6 with Klinefelter syndrome (males having karyotype XXY), and 19 with autosomal anomalies (deletion, amplification or mitotic events). Thirty-three samples were identified as having non-European Ancestry using STRUCTURE(Pritchard et al., 2000) and Eigenstrat.(Price et al., 2006) Twenty-three samples had genotype call rate <98%. In summary, 91 individuals were excluded due to the following reasons: genotype QC (n=23 with genotype call rate <98%), non-European (i.e., African or Asian) race (n=33), and relatedness (n=35). The Coriell concordance rate was 99.93%. The blind duplicate reproducibility rate was 99.99%. From a total of 657,366 SNPs, 116,857 SNPS were removed for the following reasons: monomorphic and intensity only (n=112,065), technical failures (n=1186), call rates below 0.98 (n=3467), at least one discordant call among the 58 duplicates (n=1954), and any one of at least one Mendelian error among the 12 Coriell control trios or the 9 discovered parent-offspring pairs (n=821). After these exclusions, a total of 537,872 SNPs remained for analysis. Samples with MAF <5% and HWE *P*-value $<10^{-5}$ were flagged.

Sex check: We used the PLINK option for sex check based on chromosome X heterozygosity and the intensity plot based on intensity values from chromosomes Y and X to characterize sex chromosome anomalies. We identified 6 subjects (Supplementary Figure I) with Klinefelter syndrome (top right on the plot) and two with mismatched sex (top left and bottom right on the plot)

Relatedness: We identified 8 genetically identical pairs (unintentional duplicates), 9 parent- child pairs, 17 full sibling pairs, and 12 pairs that are either grandparent- grandchild, avuncular, or half-siblings.

Population Stratification: Population stratification was performed using principal component analysis (Supplementary Figure II). Principal Components 1 (PC1) and 2 (PC2) using all 3336 eMERGE PAD samples and 209 samples from the three HAPMAP populations (green=CEU; blue=JPT/CHN; red=YRI) and 73,051 SNPs (Supplementary Figure II, Figure on the left). PC1 vs. PC2 plot using all 3336 eMERGE samples using no outlier removal in Eigensoft (Supplementary Figure II, Figure on the right). The dots lines correspond to 6 SD from the mean of the PCs. HLA SNPs were removed from the selected SNPs.

Eleven samples were excluded due to study exclusion criteria (n=2) and mislabeled samples (n=9). A further 91 were removed for genotype errors (n=23), non-European ancestry (n=33) or relatedness (n=35) (see Supplementary Data for additional details). Sex chromosome anomalies and IBD coefficients in the combined cohort (Supplementary Figure S1), population stratification using principal component analysis (Supplementary Figure S2) and flow-chart describing the study design are provided (Supplementary Figure S3).

Replication Phase

SNPs were selected based on the most significant SNPs with *P* <1xE-04. The first tier consisted of 60 candidate SNPs. Based on the final score from Illumina, SNPs were eliminated if the design score was <0.5, LD was high (>0.90) or MAF in controls was low (<0.05). After culling based on these criteria, 48 SNPs remained.

Genotyping methods:10-20 ng DNA was PCR amplified according to manufacturing instructions. End reactions were read on the ABI Prism 7900ht using ABI Sequence Detection Software (SDS)

allelic discrimination. The quality value percentage is a quality metric that indicates the reliability of called genotypes generated by the SDS software. The quality value was calculated by using ABI's proprietary calling algorithm determining how well that sample fits into the cluster. Genotypes less than 95% are located further from their clusters and have a lower reliability. An electronic data file was generated that contained genotypes and the quality value. DNA samples were assessed for quality prior to running any high throughput analysis using PicoGreen®. In all genotype assays, internal controls were used in PCR amplification in duplicate and run through the system to ensure genotyping accuracy. These included a CEPH DNA trio (parents and child) included in each 96 well plate, each in duplicate. Estimates of reproducibility were calculated periodically during the genotyping. Standard quality control procedures were applied including evaluation of sample and marker call rate, HapMap concordance, and Hardy-Weinberg in controls only. We removed 6 patients (2 male cases, 2 female cases, and 2 male controls) for low call rates (<95%).

SNP selection: The samples were plated in 21 plates using a block-randomization scheme. Individuals were selected at random by equal number of cases and controls, stratified by age and sex to avoid potential batch effect. Each plate contained 6 QC samples consisting of duplicates of 3 CEPH controls (family 1334, NA10847, NA12146, NA12239). Twenty-one participants' samples were duplicated throughout the 21 plates. Eight samples failed PicoGreen®, and they were replaced by new control samples. The plates were designed to include 6 QC samples and one sample duplicated every 2 plates.

Association of variants in *MAPK10* and *LOC388882-IGLL1* loci with PAD
In addition to genetic variant in the *ATXN2-SH2B3* locus, two additional variants in chromosomal regions 4q22.1-q23 (rs11726269) and (22q11.23) rs131408 were associated with PAD in our combined cohort.

Chromosome 4q22.1-q23: An intronic SNP in *MAPK10* (mitogen activated protein kinase 10) on chromosomal region 4q22.1-q23 was associated with PAD. SNP rs11726269 is localized to an intron of *MAPK10* on chromosome 4 had OR 1.42 and *P*-value 7.94×10^{-5} in the discovery sample, OR 1.38 and *P*-value 5.57×10^{-3} in the replication sample, and OR 1.39 and *P*-value 1.69×10^{-6} in the combined sample. *MAPK10* (also known as *JNK3*) is a member of mitogen activated protein kinase family(Muslin, 2008) plays regulatory roles in signaling pathways and acts as an integration point for multiple biochemical signals thereby influencing several cellular processes such as adhesion, proliferation, and migration.(Manning and Davis, 2003) *MAPK10* has been reported to be involved in 34 different pathways.(1995) We have previously reported that genes in nine of those pathways were differentially expressed in peripheral blood mononuclear cells (PBMC) from patients with PAD (Masud et al., 2012) and another group reported its role in modulating miRNAs differentially expressed in PBMCs of PAD patients.(Stather et al., 2013)

Chromosome 22q11.23: A SNP rs131408 in the intergenic region bounded by *LOC388882* and *IGLL1* on chromosomal region 22q11.23 was also associated with PAD in both the discovery and replication cohorts. SNP rs131408 in the intergenic region bounded by *LOC388882* and *IGLL1* on chromosome 22 had OR 1.25 and *P*-value 4.30×10^{-5} in the discovery sample, OR 1.17 and *P*-value 2.82×10^{-2} in the replication sample, and OR 1.21 and *P*-value 6.29×10^{-6} in the combined sample. *LOC388882* is an uncharacterized gene with no previously reported genotype-phenotype association. *IGLL1* is a cluster of differentiation (CD) molecule and variants in this gene have been reported to be associated with migraine(Ligthart et al., 2011) and height.(Gudbjartsson et al., 2008) Mutations in *IGLL1* have also implicated in B cell deficiency and agammaglobulinemia.(Minegishi et al., 1998)

After additional adjustment for smoking status, diabetes, diastolic blood pressure, these SNPs remained significantly associated with PAD. These different chromosomal regions were associated with PAD after excluding samples with poorly compressible vessels *(*See Supplementary Table S1; Figure S4) indicating the associations are significant for PAD.

Common pathways mediated by *SH2B3* and *MAPK10*

Angiogenic factors including VEGF, Ang-1 and eNOS where also elevated in EPCs of *Lnk*/*SH2B3*$^{-/-}$ mice. In-vitro studies in human endothelial cell lines have shown that *Lnk*/*SH2B3* interacts with integrin proteins, *ILK* and *FAK* and participate in *AKT* signaling cascade.(Devalliere and Charreau, 2011;Devalliere et al., 2012) In a prior study we found variants in endothelial nitric oxide synthase (*NOS3*) to be associated with ABI and the presence of PAD.(Kullo et al., 2008) *NOS3* plays key role in vascular tone, vascular remodeling, thrombogenicity, and platelet aggregation via nitric oxide (NO) signaling.(Fleming et al., 1999;Forstermann and Munzel, 2006) We were not able to replicate association of *NOS3* variants with PAD in the present study. However, *SH2B3* and MAPK10 play key roles in the upstream of NO signaling pathway (Sessa, 2004;Rastaldo et al., 2007;Seddon et al., 2007;Caporali and Emanueli, 2009) and neurotrophin signaling pathway and variants in these genes may impact *PI3K-AKT-NOS3* signaling cascades implicated in peripheral vascular disease.

W262R mutation in the Pleckstrin Homology domain introduces a new phosphorylation site in SH2B3

We scanned the wild type and mutant sequences of SH2B3 protein for potential sequence-level alterations using ScanProsite tool using sequence profiles of post-translational modifications sites (PTMs); wild type sequence had 31 hits and mutant sequence had 32 hits (Supplementary Figure S5). A new frequently occurring PTM site (PS00004 | CAMP_PHOSPHO_SITE, Sequence motif: RRcT (261-264)) was gained upon inducing the W262R mutation, suggesting that the threonine residue next to the mutation site (position 264) could be phosphorylated by kinases including *LCK*, a first-degree interactant of SH2B3. As a key adaptor protein involved in multiple signaling pathways, this gain of phosphorylation site could impact several downstream pathways of SH2B3.

Gene ontology enrichment analyses of SH2B3 interactome

We used experimentally validated protein interaction network of SH2B3 from STRING database. A total of seven interacting proteins were retrieved (Supplementary Figure S6); we used the list of SH2B3 and its seven interactors as input for GO term enrichment analyses. Results from GO term enrichment analyses are summarized in Table S2.

Homology modeling of Pleckstrin Homology domain of SH2B3 protein

A protein structure is a prerequisite to perform all atom-model simulations. Crystallography data for pleckstrin homology domain of SH2B3 was not available. A canonical homologous protein model of the PH domain (residues from 197 to 312; including W262R) encoded in the SH2B3 was generated using SwissModel workspace (Arnold et al., 2006). Homology model was generated from the template structure structure of the pleckstrin homology domain of mouse aps protein from Protein Data Bank. Query sequence and template sequence had a sequence identify of 44%. Qualities of the homology models were assessed using Ramachandran map / PROCHECK (Laskowski, 2009). A point mutation in the wild-type structure was introduced using PyMOL Mutagenesis Wizard (The PyMOL Molecular Graphics System, Version 1.5.0.4 Schrödinger, LLC. CA). After homology modeling, we mapped Kyte-Dolittle hydrophobicity indices (Kyte and Doolittle, 1982) onto the protein structure using Chimera (Pettersen et al., 2004). Compared to the wild type PH domain (see Figure S7(a)), substituting tryptophan with an

arginine introduces a hydrophilic patch in the W262R pleckstrin homology domain structure (see Figure S7(b))

Molecular Dynamics simulation of rs3184504 (c.784T>C) / W262R mutation in Pleckstrin Homology domain of SH2B3 protein

To understand global implications of the W262R mutation on pleckstrin homology domain structure, two 1000 picoseconds (1 nanosecond) all-atom molecular dynamic simulations were performed using GROMACS suite v4.0.7. (Van Der Spoel et al., 2005) We generated Gromacs topology files from the wild type pleckstrin homology domain and W262R pleckstrin homology domain using OPLS-AA/L force field. We defined a cubic simulation box with 0.5 nm distance was defined to add the solvent. Water molecules were added to the system using SPC water system. A preliminary energy minimization was performed for 500 steps using steepest descent algorithm and system had negative charges. Water molecules around the proteins were equilibrated after controlling the pressure and temperature of the system. Finally two production simulation runs were performed for wild type PH domain and W262R PH domains on a multi-core Linux server. The simulation trajectories were analyzed for global structural changes observed in the wild type pleckstrin homology domain and W262R pleckstrin homology domains using root mean square displacement (RMSD) observed in the trajectory over the simulations. We used g_rms program from the Gromacs suite to calculate the RMSD between initial structure and structural conformations obtained from the molecular dynamics simulations (Figure S8).

Supplementary Tables:

Table S1: Association of chromosomal regions 4q22.1-q23 *(MAPK10)* and 22q11.23 *(LOC388882-IGLL1)* with PAD

rsID (Gene symbol)	TA	Discovery cohort			Replication cohort			Combined cohort	
		**MAF**	**OR (CI)**	***P***	**MAF**	**OR (CI)**	***P***	**OR (CI)**	***P***
rs11726269 (*MAPK10*)	G	0.096	1.42 (1.19,1.69)	7.94E-05	0.093	1.378 (1.10,1.73)	0.0056	1.39 (1.22,1.59)	1.69E-06
rs131408 (*LOC388882-IGLL1*)	C	0.352	1.249 (1.12,1.39)	4.3E-05	0.350	1.169 (1.02,1.35)	0.0282	1.211 (1.11,1.32	6.29E-06

TA: Tested allele

MAF: Minor allele frequency

OR = odds ratio; CI = confidence interval (95%);

Table S2: Gene Ontology term enrichment analyses of SH2B3 protein interactome

**GO ID**	**Molecular Function Terms**	**No: of Genes***	***P*-value****
GO:0050900	Leukocyte migration	5	2.37E-04
GO:0050852	T cell receptor signaling pathway	4	1.02E-03
GO:0002768	Immune response-regulating cell surface receptor signaling pathway	4	5.22E-03
GO:0009611	Response to wounding	6	1.72E-02
GO:0007596	Blood coagulation	5	1.87E-02
GO:0050817	Coagulation	5	1.87E-02
GO:0007599	Hemostasis	5	1.92E-02
GO:0001775	Cell activation	5	2.33E-02
GO:0002764	Immune response-regulating signaling pathway	4	2.90E-02
GO:0018108	Peptidyl-tyrosine phosphorylation	3	3.34E-02
GO:0016477	Cell migration	5	3.70E-02
GO:0018212	Peptidyl-tyrosine modification	3	3.73E-02
GO:0050878	Regulation of body fluid levels	5	4.42E-02
GO:0042060	Wound healing	5	5.16E-02
GO:0048870	Cell motility	5	5.74E-02
GO:0051674	Localization of cell	5	5.74E-02
**GO ID**	**Biological Process Terms**	**No: of Genes**	***P-value***
GO:0005167	Neurotrophin TRK receptor binding	2	9.68E-03
GO:0005168	Neurotrophin TRKA receptor binding	2	9.68E-03
GO:0005165	Neurotrophin receptor binding	2	2.03E-02

*: Gene products annotated with a GO term in the interactome of SH2B3

**: Bonferroni corrected

Table S3. Pleiotropic associations of rs3184504 and rs653178 in *ATXN2*-SH2B3 locus

Disease or clinical phenotype	RAF	OR / Beta*	*P*-value*	References
Beta-2 microglubulin plasma levels	0.49	.02 [0.012-0.028] unit decrease	3E-8	(Tin et al., 2013)
Celiac disease and Rheumatoid arthritis	NR	NR	3E-19	(Zhernakova et al., 2011)
Chronic kidney disease	0.50	.01 [0.009-0.017] ml/min/1.73 m2 increase	4E-11	(Kottgen et al., 2010)
Coronary heart disease	0.44	1.07	6E-6	(Schunkert et al., 2011)
Diastolic blood pressure	0.48	.48mm Hg increase	3E-14	(Levy et al., 2009)
Diastolic blood pressure	0.47	.448 (increase)	4E-25	(Ehret et al., 2011)
Eosinophil counts	0.38	7.6 % standard unit increase	7E-19	(Gudbjartsson et al., 2009)
Hypothyroidism	0.502	1.2	3E-12	(Eriksson et al., 2012)
Hypothyroidism (PheWAS)**	0.50	1.18	1.7E-06	(Shameer et al., 2013)
Myocardial infarction	NR	1.13, 95% (CI – 1.08-1.18)	8E-8	(Gudbjartsson et al., 2009)
Myocardial infarction (PheWAS)**	0.50	1.18	1.7E-04	(Shameer et al., 2013)
Platelet count	NR	3.99(0.374)	1E-26	(Gieger et al., 2011)
Platelet count (eMERGE)	0.50	-5.33 (based on major allele C; decrease)	5E-11	(Shameer et al., 2013)
Rheumatoid arthritis	0.51	1.08	6E-6	(Stahl et al., 2010)
Systolic blood pressure	0.48	.58mm Hg increase	5E-9	(Levy et al., 2009)
Thrombotic Antiphospholipid Syndrome***	NA	Copy number variation type: Copy gain	5.4E-144	(Ochoa et al., 2013)
Type 1 diabetes	NR	NR	3E-27	(Barrett et al., 2009)
Type 1 diabetes autoantibodies	NR	1.3	2E-38	(Plagnol et al., 2011)
Urate levels	0.51	.035 [0.025-0.045] mg/dl decrease	7E-12	(Kottgen et al., 2013)

RAF: Risk allele frequency
NR: Not reported
NA: Not applicable
*: Data obtained from respective publications
**: Phenome-wide association study
***: Array-CGH and fine-mapping study

Supplementary Figures:

Figure S1: Sex chromosome anomalies and IBD coefficients in combined cohort

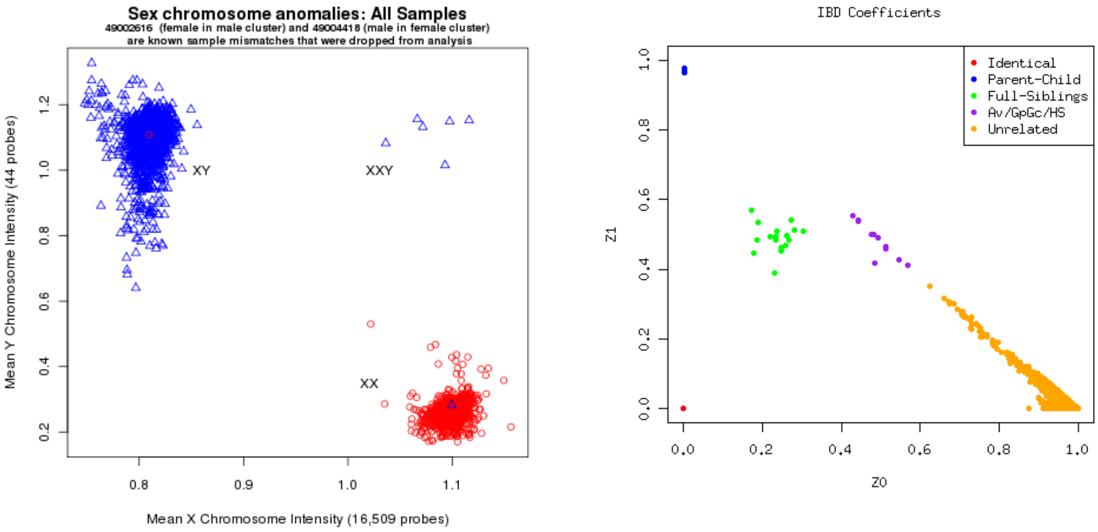

IBD coefficients (Z0 and Z1), the probabilities of sharing alleles that are identical by descent, were estimated using a method of moments procedure implemented in PLINK and plotted using R base graphics.

Figure S2: Population stratification using principal component analysis

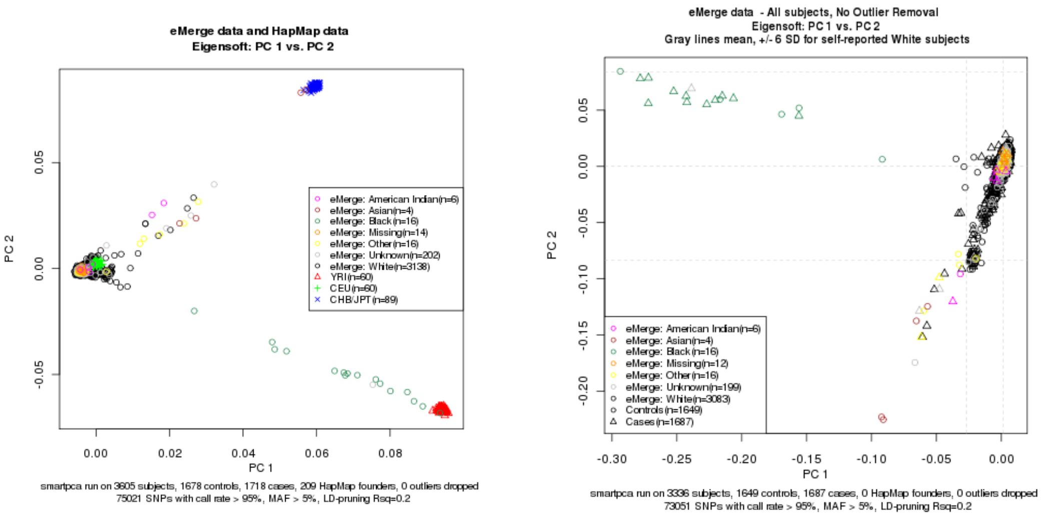

Principal Components 1 (PC1) and 2 (PC2) 3336 eMERGE PAD samples and 209 samples from the three HAPMAP populations (green=CEU; blue=JPT/CHN; red=YRI) and 73,051 SNPs b) PC1 vs. PC2 plot using all 3336 eMERGE samples using no outlier removal

Figure S3: Study design

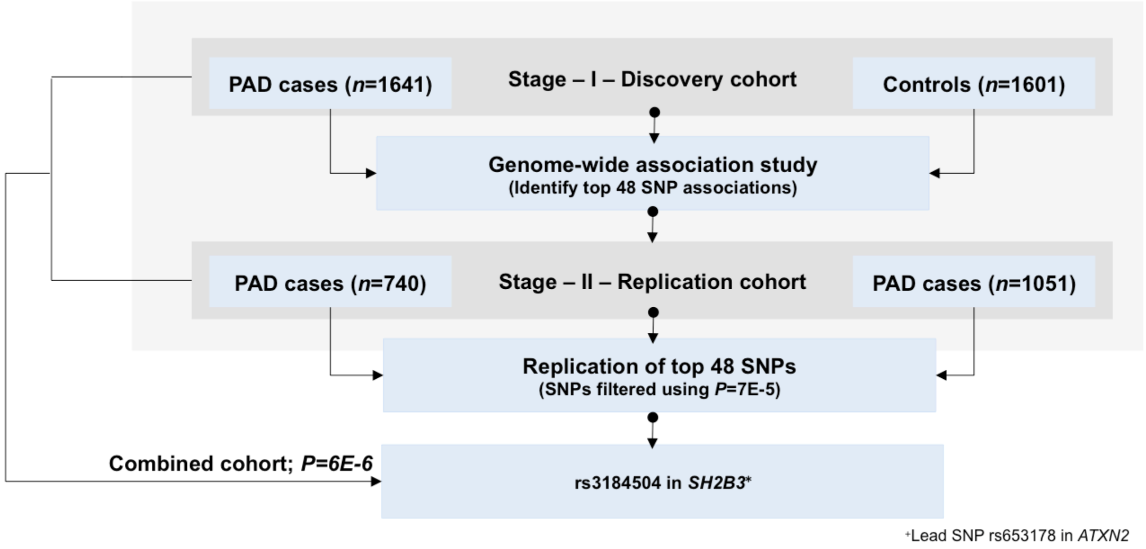

Figure S4. Visualization of chromosomal region 12q24.12 associated with PAD using discovery cohort

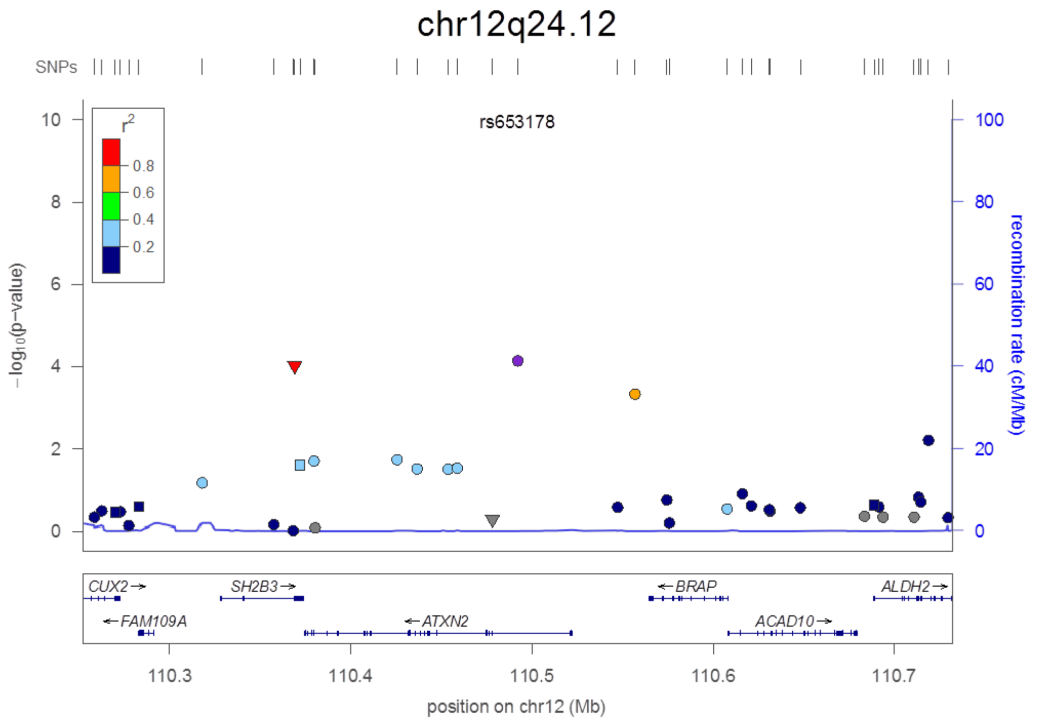

Single-nucleotide polymorphisms (SNPs) are plotted by position on the chromosome (x-axis) and the association with PLT ($-\log_{10} P$ value) on the y-axis. The rs number for the most significant SNP in the joint analysis is shown on the plot. Estimated recombination rates (from HapMap) are plotted in cyan to reflect the local linkage disequilibrium (LD) structure. The SNPs near the most significant SNP are color coded to depict their LD with the lead SNP (derived using pairwise r^2 values from the HapMap CEU data). Genes, the position of exons and the direction of transcription from the University of California, Santa Cruz genome browser are also plotted. ▾ = nonsynonymous; ○ = no annotation; ⊠ = conserved in mammals; * = conserved TFBS (transcription factor binding site). cM/Mb = centimorgan/megabase; Mb = megabase.

Figure S5: Post-translational modification sites predicted from sequence of a) SH2B3 wild type sequence and b) SH2B3 sequence with W262R mutation

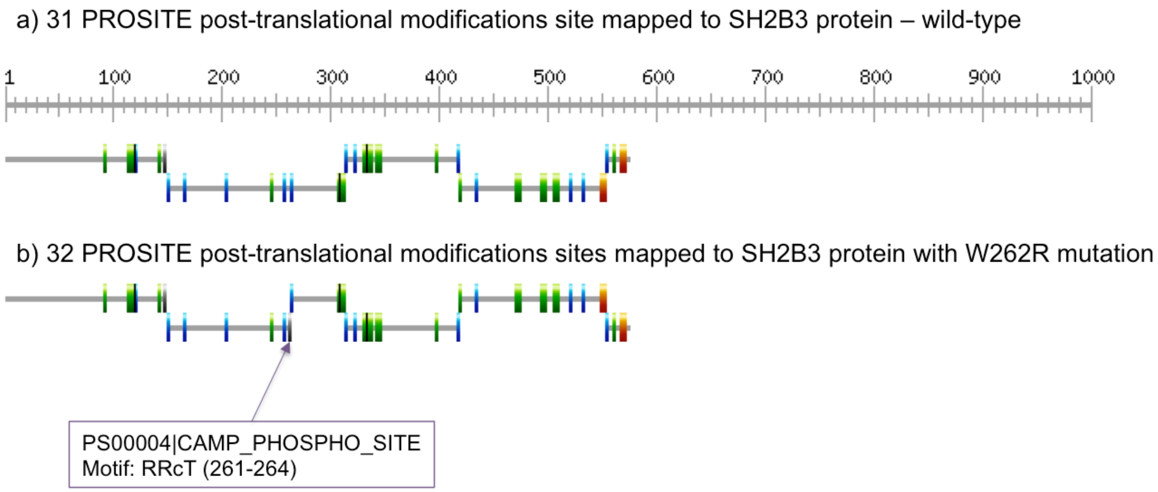

Grey vertical line indicates sequence of the protein and horizontal lines indicate one of the five post-translational modification sites (cAMP- and cGMP-dependent protein kinase phosphorylation site, protein kinase C phosphorylation site, casein kinase II phosphorylation site, tyrosine kinase phosphorylation site and N-myristoylation site).

Figure S6: Experimentally validated protein-protein interaction mediated by SH2B3

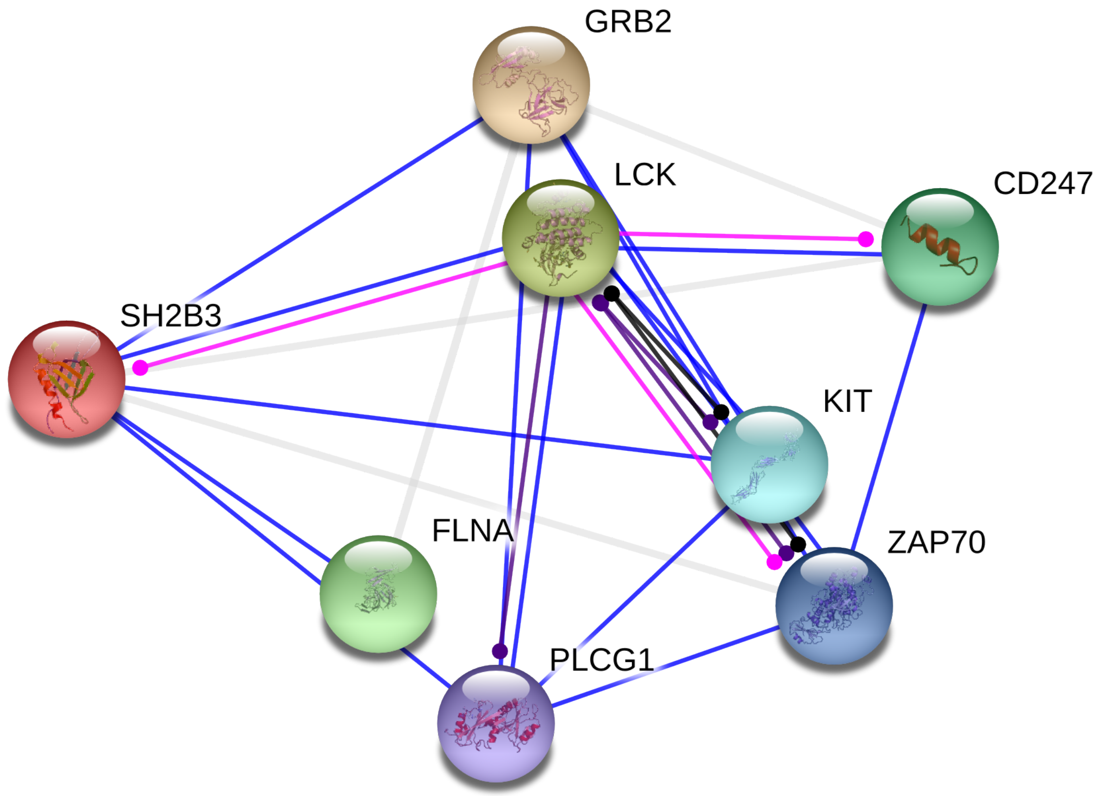

Nodes are proteins interacting with SH2B3; edges represent mode of interaction (blue: binding; purple: post-translational modifications; black: biochemical reactions; grey: other validated biochemical experiments)

Figure S7: Hydrophobicity surface renderings of a) wild type and b)W262R pleckstrin homology domains of SH2B3 protein

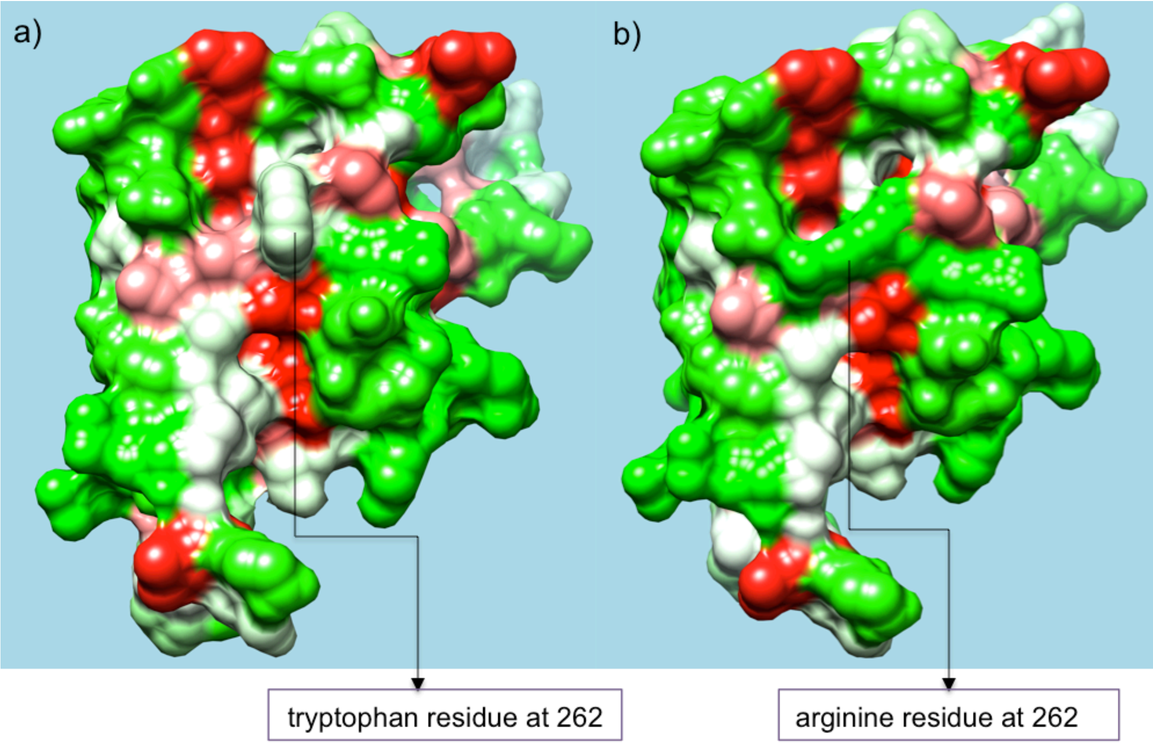

Kyte-Dolittle hydropathicity scale was mapped using a three-color scheme: minimum was defined using green; zero using white and maximum using red.

Figure S8: Root mean square deviations observed for wild type pleckstrin homology domain (black) and W262 mutant pleckstrin homology domain structures over 1000 ps molecular dynamics simulation in aqueous environment

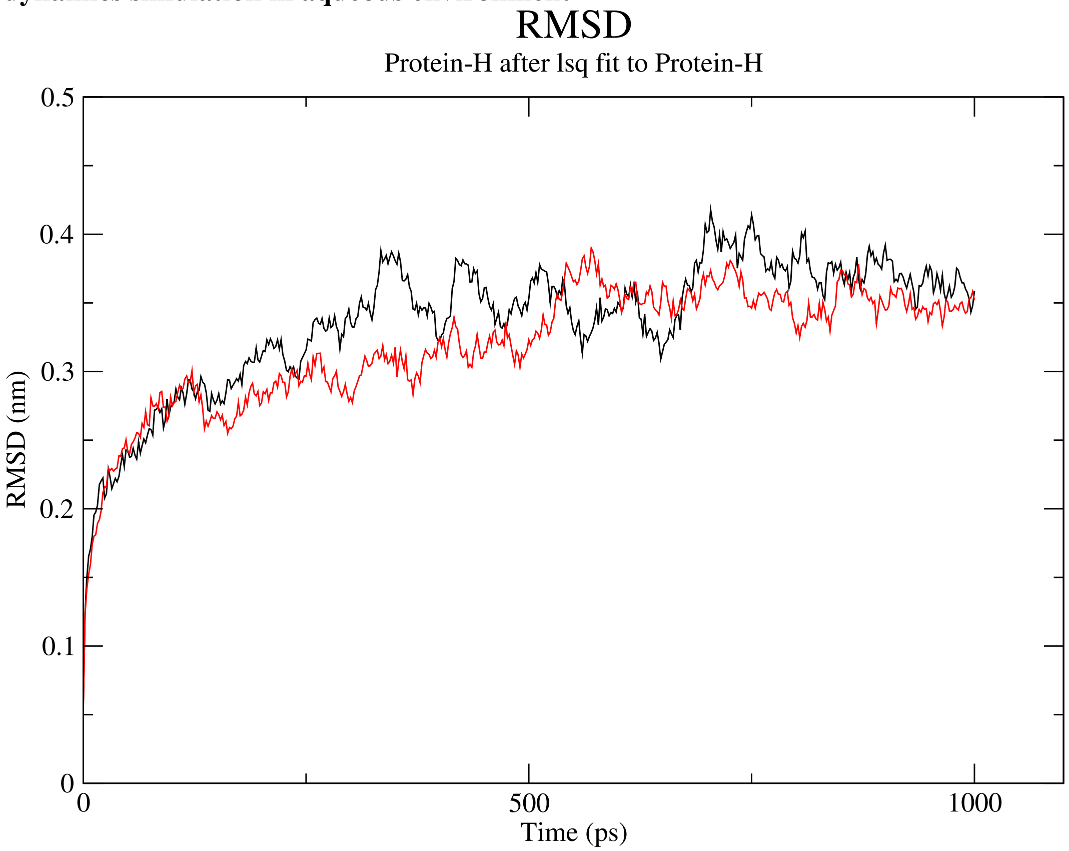

Supplementary References:

(1995). "KEGG: Kyoto Encyclopedia of Genes and Genomes". Kanehisa Laboratories).

Arnold, K., Bordoli, L., Kopp, J., and Schwede, T. (2006). The SWISS-MODEL workspace: a web-based environment for protein structure homology modelling. *Bioinformatics* 22**,** 195-201. doi: 10.1093/bioinformatics/bti770.

Barrett, J.C., Clayton, D.G., Concannon, P., Akolkar, B., Cooper, J.D., Erlich, H.A., Julier, C., Morahan, G., Nerup, J., Nierras, C., Plagnol, V., Pociot, F., Schuilenburg, H., Smyth, D.J., Stevens, H., Todd, J.A., Walker, N.M., and Rich, S.S. (2009). Genome-wide association study and meta-analysis find that over 40 loci affect risk of type 1 diabetes. *Nature genetics* 41**,** 703-707. doi: 10.1038/ng.381.

Caporali, A., and Emanueli, C. (2009). Cardiovascular actions of neurotrophins. *Physiol Rev* 89**,** 279-308. doi: 10.1152/physrev.00007.2008.

Devalliere, J., and Charreau, B. (2011). The adaptor Lnk (SH2B3): an emerging regulator in vascular cells and a link between immune and inflammatory signaling. *Biochem Pharmacol* 82**,** 1391-1402. doi: 10.1016/j.bcp.2011.06.023
S0006-2952(11)00406-0 [pii].

Devalliere, J., Chatelais, M., Fitau, J., Gerard, N., Hulin, P., Velazquez, L., Turner, C.E., and Charreau, B. (2012). LNK (SH2B3) is a key regulator of integrin signaling in endothelial cells and targets alpha-parvin to control cell adhesion and migration. *FASEB J* 26**,** 2592-2606. doi: 10.1096/fj.11-193383
fj.11-193383 [pii].

Ehret, G.B., Munroe, P.B., Rice, K.M., Bochud, M., Johnson, A.D., Chasman, D.I., Smith, A.V., Tobin, M.D., Verwoert, G.C., Hwang, S.J., Pihur, V., Vollenweider, P., O'reilly, P.F., Amin, N., Bragg-Gresham, J.L., Teumer, A., Glazer, N.L., Launer, L., Zhao, J.H., Aulchenko, Y., Heath, S., Sober, S., Parsa, A., Luan, J., Arora, P., Dehghan, A., Zhang, F., Lucas, G., Hicks, A.A., Jackson, A.U., Peden, J.F., Tanaka, T., Wild, S.H., Rudan, I., Igl, W., Milaneschi, Y., Parker, A.N., Fava, C., Chambers, J.C., Fox, E.R., Kumari, M., Go, M.J., Van Der Harst, P., Kao, W.H., Sjogren, M., Vinay, D.G., Alexander, M., Tabara, Y., Shaw-Hawkins, S., Whincup, P.H., Liu, Y., Shi, G., Kuusisto, J., Tayo, B., Seielstad, M., Sim, X., Nguyen, K.D., Lehtimaki, T., Matullo, G., Wu, Y., Gaunt, T.R., Onland-Moret, N.C., Cooper, M.N., Platou, C.G., Org, E., Hardy, R., Dahgam, S., Palmen, J., Vitart, V., Braund, P.S., Kuznetsova, T., Uiterwaal, C.S., Adeyemo, A., Palmas, W., Campbell, H., Ludwig, B., Tomaszewski, M., Tzoulaki, I., Palmer, N.D., Aspelund, T., Garcia, M., Chang, Y.P., O'connell, J.R., Steinle, N.I., Grobbee, D.E., Arking, D.E., Kardia, S.L., Morrison, A.C., Hernandez, D., Najjar, S., Mcardle, W.L., Hadley, D., Brown, M.J., Connell, J.M., Hingorani, A.D., Day, I.N., Lawlor, D.A., Beilby, J.P., Lawrence, R.W., Clarke, R., et al. (2011). Genetic variants in novel pathways influence blood pressure and cardiovascular disease risk. *Nature* 478**,** 103-109. doi: 10.1038/nature10405.

Eriksson, N., Tung, J.Y., Kiefer, A.K., Hinds, D.A., Francke, U., Mountain, J.L., and Do, C.B. (2012). Novel associations for hypothyroidism include known autoimmune risk loci. *PLoS One* 7**,** e34442. doi: 10.1371/journal.pone.0034442.

Fleming, I., Bauersachs, J., Schafer, A., Scholz, D., Aldershvile, J., and Busse, R. (1999). Isometric contraction induces the Ca2+-independent activation of the endothelial nitric oxide synthase. *Proc Natl Acad Sci U S A* 96**,** 1123-1128.

Forstermann, U., and Munzel, T. (2006). Endothelial nitric oxide synthase in vascular disease: from marvel to menace. *Circulation* 113**,** 1708-1714. doi: 10.1161/CIRCULATIONAHA.105.602532.

Gieger, C., Radhakrishnan, A., Cvejic, A., Tang, W., Porcu, E., Pistis, G., Serbanovic-Canic, J., Elling, U., Goodall, A.H., Labrune, Y., Lopez, L.M., Magi, R., Meacham, S., Okada, Y., Pirastu, N., Sorice, R., Teumer, A., Voss, K., Zhang, W., Ramirez-Solis, R., Bis, J.C., Ellinghaus, D., Gogele, M., Hottenga, J.J., Langenberg, C., Kovacs, P., O'reilly, P.F., Shin, S.Y., Esko, T., Hartiala, J., Kanoni, S., Murgia, F., Parsa, A., Stephens, J., Van Der Harst, P., Ellen Van Der Schoot, C., Allayee, H., Attwood, A., Balkau, B., Bastardot, F., Basu, S., Baumeister, S.E., Biino, G., Bomba, L., Bonnefond, A., Cambien, F., Chambers, J.C., Cucca, F., D'adamo, P., Davies, G., De Boer, R.A., De Geus, E.J., Doring, A., Elliott, P., Erdmann, J., Evans, D.M., Falchi, M., Feng, W., Folsom, A.R., Frazer, I.H., Gibson, Q.D., Glazer, N.L., Hammond, C., Hartikainen, A.L., Heckbert, S.R., Hengstenberg, C., Hersch, M., Illig, T., Loos, R.J., Jolley, J., Tee Khaw, K., Kuhnel, B., Kyrtsonis, M.C., Lagou, V., Lloyd-Jones, H., Lumley, T., Mangino, M., Maschio, A., Mateo Leach, I., Mcknight, B., Memari, Y., Mitchell, B.D., Montgomery, G.W., Nakamura, Y., Nauck, M., Navis, G., Nothlings, U., Nolte, I.M., Porteous, D.J., Pouta, A., Pramstaller, P.P., Pullat, J., Ring, S.M., Rotter, J.I., Ruggiero, D., Ruokonen, A., Sala, C., Samani, N.J., Sambrook, J., Schlessinger, D., et al. (2011). New gene functions in megakaryopoiesis and platelet formation. *Nature*. doi: nature10659 [pii]
10.1038/nature10659.

Gudbjartsson, D.F., Bjornsdottir, U.S., Halapi, E., Helgadottir, A., Sulem, P., Jonsdottir, G.M., Thorleifsson, G., Helgadottir, H., Steinthorsdottir, V., Stefansson, H., Williams, C., Hui, J., Beilby, J., Warrington, N.M., James, A., Palmer, L.J., Koppelman, G.H., Heinzmann, A., Krueger, M., Boezen, H.M., Wheatley, A., Altmuller, J., Shin, H.D., Uh, S.T., Cheong, H.S., Jonsdottir, B., Gislason, D., Park, C.S., Rasmussen, L.M., Porsbjerg, C., Hansen, J.W., Backer, V., Werge, T., Janson, C., Jonsson, U.B., Ng, M.C., Chan, J., So, W.Y., Ma, R., Shah, S.H., Granger, C.B., Quyyumi, A.A., Levey, A.I., Vaccarino, V., Reilly, M.P., Rader, D.J., Williams, M.J., Van Rij, A.M., Jones, G.T., Trabetti, E., Malerba, G., Pignatti, P.F., Boner, A., Pescollderungg, L., Girelli, D., Olivieri, O., Martinelli, N., Ludviksson, B.R., Ludviksdottir, D., Eyjolfsson, G.I., Arnar, D., Thorgeirsson, G., Deichmann, K., Thompson, P.J., Wjst, M., Hall, I.P., Postma, D.S., Gislason, T., Gulcher, J., Kong, A., Jonsdottir, I., Thorsteinsdottir, U., and Stefansson, K. (2009). Sequence variants affecting eosinophil numbers associate with asthma and myocardial infarction. *Nature genetics* 41**,** 342-347. doi: 10.1038/ng.323.

Gudbjartsson, D.F., Walters, G.B., Thorleifsson, G., Stefansson, H., Halldorsson, B.V., Zusmanovich, P., Sulem, P., Thorlacius, S., Gylfason, A., Steinberg, S., Helgadottir, A., Ingason, A., Steinthorsdottir, V., Olafsdottir, E.J., Olafsdottir, G.H., Jonsson, T., Borch-Johnsen, K., Hansen, T., Andersen, G., Jorgensen, T.,

Pedersen, O., Aben, K.K., Witjes, J.A., Swinkels, D.W., Den Heijer, M., Franke, B., Verbeek, A.L., Becker, D.M., Yanek, L.R., Becker, L.C., Tryggvadottir, L., Rafnar, T., Gulcher, J., Kiemeney, L.A., Kong, A., Thorsteinsdottir, U., and Stefansson, K. (2008). Many sequence variants affecting diversity of adult human height. *Nat Genet* 40**,** 609-615. doi: 10.1038/ng.122.

Kottgen, A., Albrecht, E., Teumer, A., Vitart, V., Krumsiek, J., Hundertmark, C., Pistis, G., Ruggiero, D., O'seaghdha, C.M., Haller, T., Yang, Q., Tanaka, T., Johnson, A.D., Kutalik, Z., Smith, A.V., Shi, J., Struchalin, M., Middelberg, R.P., Brown, M.J., Gaffo, A.L., Pirastu, N., Li, G., Hayward, C., Zemunik, T., Huffman, J., Yengo, L., Zhao, J.H., Demirkan, A., Feitosa, M.F., Liu, X., Malerba, G., Lopez, L.M., Van Der Harst, P., Li, X., Kleber, M.E., Hicks, A.A., Nolte, I.M., Johansson, A., Murgia, F., Wild, S.H., Bakker, S.J., Peden, J.F., Dehghan, A., Steri, M., Tenesa, A., Lagou, V., Salo, P., Mangino, M., Rose, L.M., Lehtimaki, T., Woodward, O.M., Okada, Y., Tin, A., Muller, C., Oldmeadow, C., Putku, M., Czamara, D., Kraft, P., Frogheri, L., Thun, G.A., Grotevendt, A., Gislason, G.K., Harris, T.B., Launer, L.J., Mcardle, P., Shuldiner, A.R., Boerwinkle, E., Coresh, J., Schmidt, H., Schallert, M., Martin, N.G., Montgomery, G.W., Kubo, M., Nakamura, Y., Munroe, P.B., Samani, N.J., Jacobs, D.R., Jr., Liu, K., D'adamo, P., Ulivi, S., Rotter, J.I., Psaty, B.M., Vollenweider, P., Waeber, G., Campbell, S., Devuyst, O., Navarro, P., Kolcic, I., Hastie, N., Balkau, B., Froguel, P., Esko, T., Salumets, A., Khaw, K.T., Langenberg, C., Wareham, N.J., Isaacs, A., Kraja, A., Zhang, Q., Wild, P.S., et al. (2013). Genome-wide association analyses identify 18 new loci associated with serum urate concentrations. *Nature genetics* 45**,** 145-154. doi: 10.1038/ng.2500.

Kottgen, A., Pattaro, C., Boger, C.A., Fuchsberger, C., Olden, M., Glazer, N.L., Parsa, A., Gao, X., Yang, Q., Smith, A.V., O'connell, J.R., Li, M., Schmidt, H., Tanaka, T., Isaacs, A., Ketkar, S., Hwang, S.J., Johnson, A.D., Dehghan, A., Teumer, A., Pare, G., Atkinson, E.J., Zeller, T., Lohman, K., Cornelis, M.C., Probst-Hensch, N.M., Kronenberg, F., Tonjes, A., Hayward, C., Aspelund, T., Eiriksdottir, G., Launer, L.J., Harris, T.B., Rampersaud, E., Mitchell, B.D., Arking, D.E., Boerwinkle, E., Struchalin, M., Cavalieri, M., Singleton, A., Giallauria, F., Metter, J., De Boer, I.H., Haritunians, T., Lumley, T., Siscovick, D., Psaty, B.M., Zillikens, M.C., Oostra, B.A., Feitosa, M., Province, M., De Andrade, M., Turner, S.T., Schillert, A., Ziegler, A., Wild, P.S., Schnabel, R.B., Wilde, S., Munzel, T.F., Leak, T.S., Illig, T., Klopp, N., Meisinger, C., Wichmann, H.E., Koenig, W., Zgaga, L., Zemunik, T., Kolcic, I., Minelli, C., Hu, F.B., Johansson, A., Igl, W., Zaboli, G., Wild, S.H., Wright, A.F., Campbell, H., Ellinghaus, D., Schreiber, S., Aulchenko, Y.S., Felix, J.F., Rivadeneira, F., Uitterlinden, A.G., Hofman, A., Imboden, M., Nitsch, D., Brandstatter, A., Kollerits, B., Kedenko, L., Magi, R., Stumvoll, M., Kovacs, P., Boban, M., Campbell, S., Endlich, K., Volzke, H., Kroemer, H.K., Nauck, M., Volker, U., Polasek, O., Vitart, V., et al. (2010). New loci associated with kidney function and chronic kidney disease. *Nature genetics* 42**,** 376-384. doi: 10.1038/ng.568.

Kullo, I.J., Greene, M.T., Boerwinkle, E., Chu, J., Turner, S.T., and Kardia, S.L. (2008). Association of polymorphisms in *NOS3* with the ankle-brachial index in hypertensive adults. *Atherosclerosis* 196**,** 905-912.

Kyte, J., and Doolittle, R.F. (1982). A simple method for displaying the hydropathic character of a protein. *Journal of molecular biology* 157**,** 105-132.

Laskowski, R.A. (2009). PDBsum new things. *Nucleic acids research* 37**,** D355-359. doi: 10.1093/nar/gkn860.

Levy, D., Ehret, G.B., Rice, K., Verwoert, G.C., Launer, L.J., Dehghan, A., Glazer, N.L., Morrison, A.C., Johnson, A.D., Aspelund, T., Aulchenko, Y., Lumley, T., Kottgen, A., Vasan, R.S., Rivadeneira, F., Eiriksdottir, G., Guo, X., Arking, D.E., Mitchell, G.F., Mattace-Raso, F.U., Smith, A.V., Taylor, K., Scharpf, R.B., Hwang, S.J., Sijbrands, E.J., Bis, J., Harris, T.B., Ganesh, S.K., O'donnell, C.J., Hofman, A., Rotter, J.I., Coresh, J., Benjamin, E.J., Uitterlinden, A.G., Heiss, G., Fox, C.S., Witteman, J.C., Boerwinkle, E., Wang, T.J., Gudnason, V., Larson, M.G., Chakravarti, A., Psaty, B.M., and Van Duijn, C.M. (2009). Genome-wide association study of blood pressure and hypertension. *Nature genetics* 41**,** 677-687. doi: 10.1038/ng.384.

Ligthart, L., De Vries, B., Smith, A.V., Ikram, M.A., Amin, N., Hottenga, J.J., Koelewijn, S.C., Kattenberg, V.M., De Moor, M.H., Janssens, A.C., Aulchenko, Y.S., Oostra, B.A., De Geus, E.J., Smit, J.H., Zitman, F.G., Uitterlinden, A.G., Hofman, A., Willemsen, G., Nyholt, D.R., Montgomery, G.W., Terwindt, G.M., Gudnason, V., Penninx, B.W., Breteler, M., Ferrari, M.D., Launer, L.J., Van Duijn, C.M., Van Den Maagdenberg, A.M., and Boomsma, D.I. (2011). Meta-analysis of genome-wide association for migraine in six population-based European cohorts. *European journal of human genetics : EJHG* 19**,** 901-907. doi: 10.1038/ejhg.2011.48.

Manning, A.M., and Davis, R.J. (2003). Targeting JNK for therapeutic benefit: from junk to gold? *Nat Rev Drug Discov* 2**,** 554-565. doi: 10.1038/nrd1132.

Masud, R., Shameer, K., Dhar, A., Ding, K., and Kullo, I.J. (2012). Gene expression profiling of peripheral blood mononuclear cells in the setting of peripheral arterial disease. *J Clin Bioinforma* 2**,** 6. doi: 10.1186/2043-9113-2-6.

Minegishi, Y., Coustan-Smith, E., Wang, Y.H., Cooper, M.D., Campana, D., and Conley, M.E. (1998). Mutations in the human lambda5/14.1 gene result in B cell deficiency and agammaglobulinemia. *The Journal of experimental medicine* 187**,** 71-77.

Muslin, A.J. (2008). MAPK signalling in cardiovascular health and disease: molecular mechanisms and therapeutic targets. *Clin Sci (Lond)* 115**,** 203-218. doi: 10.1042/CS20070430.

Ochoa, E., Iriondo, M., Bielsa, A., Ruiz-Irastorza, G., Estonba, A., and Zubiaga, A.M. (2013). Thrombotic antiphospholipid syndrome shows strong haplotypic association with SH2B3-ATXN2 locus. *PLoS One* 8**,** e67897. doi: 10.1371/journal.pone.0067897.

Pettersen, E.F., Goddard, T.D., Huang, C.C., Couch, G.S., Greenblatt, D.M., Meng, E.C., and Ferrin, T.E. (2004). UCSF Chimera--a visualization system for exploratory research and analysis. *Journal of computational chemistry* 25**,** 1605-1612. doi: 10.1002/jcc.20084.

Plagnol, V., Howson, J.M., Smyth, D.J., Walker, N., Hafler, J.P., Wallace, C., Stevens, H., Jackson, L., Simmonds, M.J., Bingley, P.J., Gough, S.C., and Todd, J.A.

(2011). Genome-wide association analysis of autoantibody positivity in type 1 diabetes cases. *PLoS genetics* 7**,** e1002216. doi: 10.1371/journal.pgen.1002216.

Price, A.L., Patterson, N.J., Plenge, R.M., Weinblatt, M.E., Shadick, N.A., and Reich, D. (2006). Principal components analysis corrects for stratification in genome-wide association studies. *Nat Genet* 38**,** 904-909. doi: ng1847 [pii]

10.1038/ng1847.

Pritchard, J.K., Stephens, M., Rosenberg, N.A., and Donnelly, P. (2000). Association mapping in structured populations. *Am J Hum Genet* 67**,** 170-181. doi: S0002-9297(07)62442-2 [pii]

10.1086/302959.

Purcell, S., Neale, B., Todd-Brown, K., Thomas, L., Ferreira, M.A., Bender, D., Maller, J., Sklar, P., De Bakker, P.I., Daly, M.J., and Sham, P.C. (2007). PLINK: a tool set for whole-genome association and population-based linkage analyses. *Am J Hum Genet* 81**,** 559-575.

Rastaldo, R., Pagliaro, P., Cappello, S., Penna, C., Mancardi, D., Westerhof, N., and Losano, G. (2007). Nitric oxide and cardiac function. *Life Sci* 81**,** 779-793. doi: 10.1016/j.lfs.2007.07.019.

Schunkert, H., Konig, I.R., Kathiresan, S., Reilly, M.P., Assimes, T.L., Holm, H., Preuss, M., Stewart, A.F., Barbalic, M., Gieger, C., Absher, D., Aherrahrou, Z., Allayee, H., Altshuler, D., Anand, S.S., Andersen, K., Anderson, J.L., Ardissino, D., Ball, S.G., Balmforth, A.J., Barnes, T.A., Becker, D.M., Becker, L.C., Berger, K., Bis, J.C., Boekholdt, S.M., Boerwinkle, E., Braund, P.S., Brown, M.J., Burnett, M.S., Buysschaert, I., Carlquist, J.F., Chen, L., Cichon, S., Codd, V., Davies, R.W., Dedoussis, G., Dehghan, A., Demissie, S., Devaney, J.M., Diemert, P., Do, R., Doering, A., Eifert, S., Mokhtari, N.E., Ellis, S.G., Elosua, R., Engert, J.C., Epstein, S.E., De Faire, U., Fischer, M., Folsom, A.R., Freyer, J., Gigante, B., Girelli, D., Gretarsdottir, S., Gudnason, V., Gulcher, J.R., Halperin, E., Hammond, N., Hazen, S.L., Hofman, A., Horne, B.D., Illig, T., Iribarren, C., Jones, G.T., Jukema, J.W., Kaiser, M.A., Kaplan, L.M., Kastelein, J.J., Khaw, K.T., Knowles, J.W., Kolovou, G., Kong, A., Laaksonen, R., Lambrechts, D., Leander, K., Lettre, G., Li, M., Lieb, W., Loley, C., Lotery, A.J., Mannucci, P.M., Maouche, S., Martinelli, N., Mckeown, P.P., Meisinger, C., Meitinger, T., Melander, O., Merlini, P.A., Mooser, V., Morgan, T., Muhleisen, T.W., Muhlestein, J.B., Munzel, T., Musunuru, K., Nahrstaedt, J., Nelson, C.P., Nothen, M.M., Olivieri, O., et al. (2011). Large-scale association analysis identifies 13 new susceptibility loci for coronary artery disease. *Nature genetics* 43**,** 333-338. doi: 10.1038/ng.784.

Seddon, M., Shah, A.M., and Casadei, B. (2007). Cardiomyocytes as effectors of nitric oxide signalling. *Cardiovascular research* 75**,** 315-326. doi: 10.1016/j.cardiores.2007.04.031.

Sessa, W.C. (2004). eNOS at a glance. *J Cell Sci* 117**,** 2427-2429. doi: 10.1242/jcs.01165.

Shameer, K., Denny, J.C., Ding, K., Jouni, H., Crosslin, D.R., De Andrade, M., Chute, C.G., Peissig, P., Pacheco, J.A., Li, R., Bastarache, L., Kho, A.N., Ritchie, M.D., Masys, D.R., Chisholm, R.L., Larson, E.B., Mccarty, C.A., Roden, D.M., Jarvik, G.P., and Kullo, I.J. (2013). A genome- and phenome-wide association study to

identify genetic variants influencing platelet count and volume and their pleiotropic effects. *Hum Genet*. doi: 10.1007/s00439-013-1355-7.

Stahl, E.A., Raychaudhuri, S., Remmers, E.F., Xie, G., Eyre, S., Thomson, B.P., Li, Y., Kurreeman, F.A., Zhernakova, A., Hinks, A., Guiducci, C., Chen, R., Alfredsson, L., Amos, C.I., Ardlie, K.G., Barton, A., Bowes, J., Brouwer, E., Burtt, N.P., Catanese, J.J., Coblyn, J., Coenen, M.J., Costenbader, K.H., Criswell, L.A., Crusius, J.B., Cui, J., De Bakker, P.I., De Jager, P.L., Ding, B., Emery, P., Flynn, E., Harrison, P., Hocking, L.J., Huizinga, T.W., Kastner, D.L., Ke, X., Lee, A.T., Liu, X., Martin, P., Morgan, A.W., Padyukov, L., Posthumus, M.D., Radstake, T.R., Reid, D.M., Seielstad, M., Seldin, M.F., Shadick, N.A., Steer, S., Tak, P.P., Thomson, W., Van Der Helm-Van Mil, A.H., Van Der Horst-Bruinsma, I.E., Van Der Schoot, C.E., Van Riel, P.L., Weinblatt, M.E., Wilson, A.G., Wolbink, G.J., Wordsworth, B.P., Wijmenga, C., Karlson, E.W., Toes, R.E., De Vries, N., Begovich, A.B., Worthington, J., Siminovitch, K.A., Gregersen, P.K., Klareskog, L., and Plenge, R.M. (2010). Genome-wide association study meta-analysis identifies seven new rheumatoid arthritis risk loci. *Nature genetics* 42**,** 508-514. doi: 10.1038/ng.582.

Stather, P.W., Sylvius, N., Wild, J.B., Choke, E., Sayers, R.D., and Bown, M.J. (2013). Differential MicroRNA Expression Profiles in Peripheral Arterial Disease. *Circ Cardiovasc Genet*. doi: CIRCGENETICS.111.000053 [pii]
10.1161/CIRCGENETICS.111.000053.

Tin, A., Astor, B.C., Boerwinkle, E., Hoogeveen, R.C., Coresh, J., and Kao, W.H. (2013). Genome-wide association study identified the human leukocyte antigen region as a novel locus for plasma beta-2 microglobulin. *Human genetics* 132**,** 619-627. doi: 10.1007/s00439-013-1274-7.

Van Der Spoel, D., Lindahl, E., Hess, B., Groenhof, G., Mark, A.E., and Berendsen, H.J. (2005). GROMACS: fast, flexible, and free. *Journal of computational chemistry* 26**,** 1701-1718. doi: 10.1002/jcc.20291.

Zhernakova, A., Stahl, E.A., Trynka, G., Raychaudhuri, S., Festen, E.A., Franke, L., Westra, H.J., Fehrmann, R.S., Kurreeman, F.A., Thomson, B., Gupta, N., Romanos, J., Mcmanus, R., Ryan, A.W., Turner, G., Brouwer, E., Posthumus, M.D., Remmers, E.F., Tucci, F., Toes, R., Grandone, E., Mazzilli, M.C., Rybak, A., Cukrowska, B., Coenen, M.J., Radstake, T.R., Van Riel, P.L., Li, Y., De Bakker, P.I., Gregersen, P.K., Worthington, J., Siminovitch, K.A., Klareskog, L., Huizinga, T.W., Wijmenga, C., and Plenge, R.M. (2011). Meta-analysis of genome-wide association studies in celiac disease and rheumatoid arthritis identifies fourteen non-HLA shared loci. *PLoS genetics* 7**,** e1002004. doi: 10.1371/journal.pgen.1002004.
